# Supplementary material for: The Dual Prey-Inactivation Strategy of Spiders—In-Depth Venomic Analysis of Cupiennius salei
Source: Toxins (Basel). 2019 Mar 19;11(3):167. doi: 10.3390/toxins11030167 (PMC6468893; doi:10.3390/toxins11030167)
Supplement: Supplementary file 1 [file toxins-11-00167-s001.zip › Supplementary Fig S1.1-S1.19/Supplementary Fig S1.12.pdf]

Supplementary Fig S1.12
CLUSTAL O(1.2.4) multiple sequence alignment: Hyaluronidases

```
tr_A0A0S4JYH2_CUPSA         ------METRIIFLHLLAI--------VSGFKIYWNVPTFQCTHNYKIDYVKLLSTYGIQ
HYAL_ALOMA                  ------MESRIIFLHLLAI--------VSGFQIYWNAPTGQCSRHYKMNFVQLLSTYGIQ
HYAL_VIRFA                  ------MTISILFCLYLGI--------ASGFEIYWNVPTAQCSGNYQIDFVSLLRTYGIR
gi_XM_016067004_PARTE       MNTLKRALCNFLYFTLLQS--------VTSFEVYWNVPTQQCLRNYGHDFTRNLKTYGIL
tr_J9XYC6_BRAVA             -------------------------KDPQVFAVRWNVPTIQCRKTYGMDFVPLLKSYGIL
tr_A0A1D0C0T3_HADIN         -------MAVFVLLLLLSTVTSQAEEDPTVFNVRWNVPTIQCLKTYGIDFVPLLRSYGIL
                                                          * : **.** **   *  ::.  * :***

tr_A0A0S4JYH2_CUPSA         VNDGGKFQGNQVTIFYETQLGLYPRILKSGKMENGGIPQRGNFEKHLEKASTDLQKVIPW
HYAL_ALOMA                  VNDGDKFQGNRVTIFYDTQLGLYPKIRKSGKMENGGIPQRGDLEQHLAKASSDLAKVIPW
HYAL_VIRFA                  VNSGDKFKGDSVTIFYEAQLGLYPRILKSGKLENGGIPQKGDLTAHLRKASKDLEKVIPW
gi_XM_016067004_PARTE       VNNGDKFQGEQITIFYESQLGFYPSILKNGTWVNGGLPQAANISKHLQRAAEDLQNTIKN
tr_J9XYC6_BRAVA             VNSEDEFKGEVNTIFYEGQLGLYPHLDQSGQRVNGGIPQLGDLPEHLKKAREDINKAIPD
tr_A0A1D0C0T3_HADIN         VNNGDEFRGGVNTIFYESQLGLYPHLNQTDYPVNGGIPQLGDLSAHLKKSRQDIDKIIPD
                            **. .:*:*   ****: ***:** : :..   ***:** .::  ** ::  *: : *

tr_A0A0S4JYH2_CUPSA         KEFSGLGVIDWEAWRPTWEFNWEPLRIYQTESIKRAKELHPTANDSAVKEIAERQWEDSA
HYAL_ALOMA                  PDFDGLGVIDWEAWRPTWEFNWEPLRIYQTESIQFVKEMYPDAKDPFLKETAERQWENST
HYAL_VIRFA                  KDFSGLGVIDWEAWRPTWEFNWGPLKIYQTESIELAKELNPTANASVIQEIAQNQWMHTA
gi_XM_016067004_PARTE       ESFNGIGVIDWEAWRPSWNFNWIPLDIYREKSREFARKQSSTLNDKEIEELAKHQWGNGA
tr_J9XYC6_BRAVA             VNFNGLGIIDWESWRPVWNFNWGALKKYQDESFEEALKQHPGRTNDSLWQLAQQEWETSA
tr_A0A1D0C0T3_HADIN         VQFTGLGIIDWESWRAVWDFNWGTLKKYQEESFRVARDQHPDWTNSSLLKSAQEEWETSA
                             .* *:*:****:**  *:***  *  *: :* . . .     .   : : *:.:*   :

tr_A0A0S4JYH2_CUPSA         KLYMLETLRLAKKLRPQAPWCYYLFPDCYNYVGKKPKDFQCSASIRKGNDKLSWLWKDST
HYAL_ALOMA                  KSYMLETLRLAKKLRPRAPWCYYLFPDCYNYVGKKPKDFQCSAAIRKGNDKLEWLWKEST
HYAL_VIRFA                  KLYMLETLRLAKKSRPKARWGYYLFPDCYNYVGKKPKDFQCSASIRKGNDKLSWLWEESS
gi_XM_016067004_PARTE       RSYMLETLELSKKLRPSANWGYYLFPDCYNYVGKKPADFECSEKVKIENDKLSWLWNSST
tr_J9XYC6_BRAVA             KNFMLETLRLAQTMRPNSLWCYYLFPDCYNYNGQTPQEFRCPSIVVTGNNQLSWLWHESK
tr_A0A1D0C0T3_HADIN         KNYMLKTLQLAQMMRPQALWCYYLYPDCYNYKGQTPQEFQCPPIVIKGNNQLSWLWDEIK
                            : :**:**.*::  ** : * ***:****** *:.* :*.*   :   *::*.***.. .

tr_A0A0S4JYH2_CUPSA         ALCPSIYVYESQLDRYSFEQRTWRDNEKLREALRVATRTSKIYPYVNYFD---K-ELIPE
HYAL_ALOMA                  AVCPSIYVYENALVQYSLDQRSWRDNEKLREAVRVASRTAKIYPYVNYFD---T-ELVPE
HYAL_VIRFA                  ALCPSIYVYESQLDRYSLEQRTWRDNEKLRETLRVASPKAKIFPYVNYFD---T-KLIPE
gi_XM_016067004_PARTE       GIFPSIYLYDNHLDHYTMEQRSYKDHEKLKEAIRVAPAEAKILPYINFHL---KKILVPQ
tr_J9XYC6_BRAVA             AVCPSLYVADGYLQKYTFEQRTWYVDGRLKEALRVAPN-SQLYPYVGYGYGVTPGAMVPE
tr_A0A1D0C0T3_HADIN         AVCPSLYVVGGYLEKYSFDQRTWYVNGRLQEALRVAPN-SQLYPYIGYGYGITPGAMVPK
                            .: **:*:  . * :*:::**::  . :*:*::***   ::: **:.:        ::*:

tr_A0A0S4JYH2_CUPSA         QEVWRMLAQAAAVGGSGAVIWGSSAAVASEELCKSLKQYIIETLGPAAEKVAWRSDLCSK
HYAL_ALOMA                  QEMWRMLAQAAAVGSSGAVIWGASSSVRSEQHCKSLKQYIIDTLGPAAEKVAWRCNLCSQ
HYAL_VIRFA                  QEMWRVLAQAAATGSDGAVIWGASASVSSENHCKNLKKYIIDTLGPAAEKVSWRSDLCSK
gi_XM_016067004_PARTE       GDFERMAAQAAAIGADGFIIWGSSSAVSSRTHCNEVNEYITQVLGPAVKKISRNAELCSR
tr_J9XYC6_BRAVA             DDFWRILAQVASAGSSGTVIWGASATLRSRDNCQLLQQYVKDILGPSVKTVKENAERCAK
tr_A0A1D0C0T3_HADIN         DDFW--------------------------------------------------------
                             :.

tr_A0A0S4JYH2_CUPSA         EICNNQGRCTFPDDDYANAWKLFTDDT-VKFYAGNITCRCSENYSGRFCEKKN-------
HYAL_ALOMA                  RICNNRGLCTFPGDDDAKAWKLFTDDT-VPFYAGNITCRCSKNYTGRFCQQ---------
HYAL_VIRFA                  QICNGRGRCTFPNDEYAKAWKLFTDDSEKKFYAGDITCRCSENYSGQFCDK---------
gi_XM_016067004_PARTE       TVCHGNGHCTWPNDKTVTAWKEFLKEY-PSFYKNEIVCKCKILYTGTFCGHQLNKLPNQ-
tr_J9XYC6_BRAVA             TVCNGKGRCTWPNDPNVVAWRVYLDRNKHPFQRSEITCHCVEGYSGRYCDVKSGITNQTK
tr_A0A1D0C0T3_HADIN         ------------------------------------------------------------

tr_A0A0S4JYH2_CUPSA         -------------------
HYAL_ALOMA                  -------------------
HYAL_VIRFA                  -------------------
```

```
gi_XM_016067004_PARTE        -------------------
tr_J9XYC6_BRAVA              RVSFKLSELYTYLRRLLDN
tr_A0A1D0C0T3_HADIN          -------------------
```

Percent Identity Matrix - created by Clustal2.1

1:	tr_A0A0S4JYH2_CUPSA	100.00	79.85	77.30	51.78	49.74	50.35
2:	HYAL_ALOMA	79.85	100.00	71.28	50.51	48.94	48.94
3:	HYAL_VIRFA	77.30	71.28	100.00	54.26	53.36	50.71
4:	gi_XM_016067004_PARTE	51.78	50.51	54.26	100.00	45.48	44.21
5:	tr_J9XYC6_BRAVA	49.74	48.94	53.36	45.48	100.00	77.34
6:	tr_A0A1D0C0T3_HADIN	50.35	48.94	50.71	44.21	77.34	100.00

Proteomic coverage trypsin

>tr|A0A0S4JYH2|A0A0S4JYH2_CUPSA Hyaluronidase OS=Cupiennius salei OX=6928 GN=Hyal PE=2 SV=1

METRIIFLHL LAIVSG**FKIY WNVPTFQCTH NYKIDYVKLL STYGIQVNDG GKFQGNQVTI FYETQLGLYP RILKSGKMEN**
GGIPQRGNFE KHLEKASTDL QKVIPWKEFS GLGVIDWEAW RPTWEFNWEP LRIYQTESIK RAKELHPTAN DSAVKEIAER
QWEDSAKLYM LETLRLAKKL RPQAPWCYYL FPDCYNYVGK KPKDFQCSAS IRKGNDKLSW LWKDSTALCP SIYVYESQLD
RYSFEQRTWR DNEKLREALR VATR**TSKIYP YVNYFDKELI PEQEVWRMLA QAAAVGGSGA VIWGSSAAVA SEELCKSLKQ**
YIIETLGPAA EKVAWRSDLC SKEICNNQGR CTFPDDDYAN AWKLFTDDTV KFYAGNITCR CSENYSGRFC EKKN

Proteomic coverage chymotrypsin

>tr|A0A0S4JYH2|A0A0S4JYH2_CUPSA Hyaluronidase OS=Cupiennius salei OX=6928 GN=Hyal PE=2 SV=1

METRIIFLHL LAIVSGFKIY **WNVPTFQCTH NYKIDYVKLL** STY**GIQVNDG GKFQGNQVTI FYETQLGLYP RILKSGKMEN**
GGIPQRGNFE KHLEKASTDL QKVIPWKEFS GLGVIDWEAW RPTWEFNWEP L**RIYQTESIK RAKEL**HPTAN DSAVKEIAER
Q**WEDSAKLYM LETLRLAKKL RPQAPW**CY**YL FPDCYNYVGK KPKDFQCSAS IRKGNDKLSW** L**WKDSTALCP SIYVYESQLD**
RYSFEQRTWR DNEKLREALR VATRTSKIYP YVNYFDKELI PEQEVWRMLA QAAAVGGSGA VIWGSSAAVA SEELCKSLKQ
YIIETLGPAA EKVAWRSDLC SKEICNNQGR CTFPDDDYAN AWKLFTDDTV KFYAGNITCR CSENYSGRFC EKKN

Supplementary Fig S1.12 Multiple sequence alignment of HYAL from the venom glands of *C. salei* (tr_A0A0S4JYH2_CUPSA), *A. marikovskyi* (HYAL_ALOMA), *V. fasciatus* (HYAL_VIRFA), *P. tepidariorum* (gi_XM_016067004_PARTE), *Brachypelma vagans* (tr_J9XYC6_BRAVA), and *Hadronyche infensa* (tr_A0A1D0C0T3_HADIN). Signal peptides are marked in black characters, cysteines in white with a black box, positively charged amino acids in pink, negatively charged amino acids in blue, hydrophobic amino acids in red and polar amino acids in green.
